# Overcoming resistance to single-agent therapy for oncogenic *BRAF* gene fusions *via* combinatorial targeting of MAPK and PI3K/mTOR signaling pathways

**DOI:** 10.18632/oncotarget.20949

**Published:** 2017-09-15

**Authors:** Payal Jain, Amanda Silva, Harry J. Han, Shih-Shan Lang, Yuankun Zhu, Katie Boucher, Tiffany E. Smith, Aesha Vakil, Patrick Diviney, Namrata Choudhari, Pichai Raman, Christine M. Busch, Tim Delaney, Xiaodong Yang, Aleksandra K. Olow, Sabine Mueller, Daphne Haas-Kogan, Elizabeth Fox, Phillip B. Storm, Adam C. Resnick, Angela J. Waanders

**Affiliations:** ^1^ Division of Neurosurgery, The Children’s Hospital of Philadelphia, Philadelphia, PA, USA; ^2^ Department of Neurosurgery, University of Pennsylvania Perelman School of Medicine, Philadelphia, PA, USA; ^3^ Center for Data Driven Discovery in Biomedicine (D3b), The Children’s Hospital of Philadelphia, Philadelphia, PA, USA; ^4^ The Fred Hutchinson Cancer Research Center, Seattle, WA, USA; ^5^ Division of Neurology, The Children’s Hospital of Philadelphia, Philadelphia, PA, USA; ^6^ Department of Biomedical and Health Informatics, The Children’s Hospital of Philadelphia, Philadelphia, PA, USA; ^7^ Center for Childhood Cancer Research, Children’s Hospital of Philadelphia, Philadelphia, PA, USA; ^8^ Division of Oncology, Department of Pediatrics, The Children’s Hospital of Philadelphia, Philadelphia, PA, USA; ^9^ Division of Neurology, University of California, San Francisco, CA, USA; ^10^ Amgen, South San Francisco, CA, USA; ^11^ Department of Neurosurgery, University of California, San Francisco, CA, USA; ^12^ Department of Pediatrics, University of California, San Francisco, CA, USA; ^13^ Department of Radiation Oncology, Harvard Medical School, Boston, MA, USA; ^14^ Department of Pediatrics, Perelman School of Medicine at the University of Pennsylvania, Philadelphia, PA, USA

**Keywords:** pediatric low-grade glioma, BRAF-fusions, trametinib and everolimus, MAPK pathway, PI3K/mTOR pathway

## Abstract

Pediatric low-grade gliomas (PLGGs) are frequently associated with activating *BRAF* gene fusions, such as KIAA1549-BRAF, that aberrantly drive the mitogen activated protein kinase (MAPK) pathway. Although RAF inhibitors (RAFi) have been proven effective in BRAF-V600E mutant tumors, we have previously shown how the KIAA1549-BRAF fusion can be paradoxically activated by RAFi. While newer classes of RAFi, such as PLX8394, have now been shown to inhibit MAPK activation by KIAA1549-BRAF, we sought to identify alternative MAPK pathway targeting strategies using clinically relevant MEK inhibitors (MEKi), along with potential escape mechanisms of acquired resistance to single-agent MAPK pathway therapies. We demonstrate effectiveness of multiple MEKi against diverse BRAF-fusions with novel N-terminal partners, with trametinib being the most potent. However, resistance to MEKi or PLX8394 develops *via* increased RTK expression causing activation of PI3K/mTOR pathway in BRAF-fusion expressing resistant clones. To circumvent acquired resistance, we show potency of combinatorial targeting with trametinib and everolimus, an mTOR inhibitor (mTORi) against multiple BRAF-fusions. While single-agent mTORi and MEKi PLGG clinical trials are underway, our study provides preclinical rationales for using MEKi and mTORi combinatorial therapy to stave off or prevent emergent drug-resistance in BRAF-fusion driven PLGGs.

## INTRODUCTION

Pediatric low-grade gliomas (PLGGs) comprise a heterogeneous group of World Health Organization (WHO) grade I and II tumors [1] and are the most common type of brain tumor in children [2]. While mortality from PLGGs remains low, cytotoxic chemotherapy and/or radiation used in treating PLGGs can lead to significant life-long neurocognitive and systemic complications [3], thereby necessitating the identification of novel targeted therapies with reduced morbidity. The characterization of *KIAA1549-BRAF* gene fusions as a common structural alteration in pilocytic astrocytomas (Grade I PLGG, PAs) provided the first genomic underpinning for PLGG pathogenesis [4, 5]. BRAF is a serine-threonine kinase that activates the highly conserved mitogen-activated protein kinase (MAPK) signaling cascade. Subsequent studies found *BRAF* mutations in nearly 80% of PAs and up to 60% of pleomorphic xanthoastrocytomas (PXAs) and gangliogliomas [6, 7]. Recent large-scale sequencing studies have identified additional BRAF-fusions with novel fusion partners in PLGGs [8-10]. In addition, BRAF-fusions occur in a wide range of adult malignancies including melanoma, gastric, thyroid, pancreatic, prostate and lung cancers [11]. A comprehensive genomic profiling study across 12 different cancer types identified BRAF-fusions in 0.3% of the 20,573 pediatric and adult tumors [12]. Furthermore, specific BRAF-fusions are common across multiple adult and pediatric cancers, including *KIAA1549-BRAF* found in pediatric gliomas, breast carcinoma, and sarcomas, and *MKRN1-BRAF* in PLGGS, colorectal carcinoma, and head and neck carcinoma [11, 12]. Together, these findings highlight the prevalence of pan-cancer BRAF-fusions.

The development of BRAF-targeted therapies was initiated with the discovery of *BRAF-V600E* point mutation in melanomas, which emerged to be one of the most prevalent *RAF* mutation across human cancers [13]. Subsequent efforts led to FDA-approval of BRAF inhibitors (BRAFi), vemurafenib and dabrafenib, against BRAF-V600E metastatic melanoma [14, 15]. However, BRAF-targeted therapies have shown diverse clinical responses to different activating *BRAF* alterations in melanoma and other cancers [16-18]. We have previously shown that unlike BRAF-V600E, KIAA1549-BRAF fusions functions as distinct, constitutive homodimers that are resistant to first-generation RAFi, vemurafenib (research analog PLX4720) and undergo paradoxical activation in response to PLX4720 [4]. In contrast, the second-generation “paradox breaking” BRAFi (PLX8394), under clinical development, was found to successfully inhibit PLGG-associated BRAF-fusion [4] and BRAF-mutant lung cancer [19]. However, a clinical trial utilizing sorafenib, a multikinase inhibitor initially developed as an allosteric BRAFi, was halted due to unexpected acceleration of PLGG tumor growth in treated children [20]. As such, research focus has moved towards targeting downstream pathway components, such as MEK, in BRAF-mutant PLGGs and adult cancers [19, 21-23]. Potent MEK inhibitors (MEKi) are FDA-approved to treat melanoma and currently being tested for advanced cancers. However, as has been repeatedly demonstrated in other cancers, even when initially successful, emergent resistance to a single-agent MAPK pathway inhibitors will likely prevail [18, 24] as patients might relapse despite treatment with single-agent targeted inhibitors. This indicates the need to understand mechanisms of resistance towards MAPK targeted therapies in order to identify rational combinatorial treatments.

PLGG-directed treatment advances have been impeded by the paucity of representative PLGG patient-derived cell lines, insufficient molecular characterization of primary tumors, and the high burden of proof needed to test novel therapies in children. To address this in our study, we characterized the sensitivity of a panel of BRAF fusions with distinct N-terminal partners to clinically relevant and mechanistically distinct MEK inhibitors (MEKi) in heterologous cell and animal model systems that have previously been predictive of clinical responses [20]. Despite several clinical trials that have begun to test targeted therapies in pediatric glioma patients with diverse mutational landscapes (clinicaltrials.gov Identifiers- NCT00782626, NCT01158651, NCT02124772, NCT01089101 and NCT01748149), there are no existing biological studies to delineate potential acquired resistance mechanisms to such therapies. We address such critical emergent questions by interrogating escape mechanisms of drug resistance to single-agent MAPK pathway-directed therapies. Finally, we identify combinatorial targeting opportunities for selective and sustained inhibition of BRAF-fusion signaling.

## RESULTS

### BRAF-fusion driven MAPK signaling pathway can be differentially inhibited by MEK inhibitors

**Figure 1:**
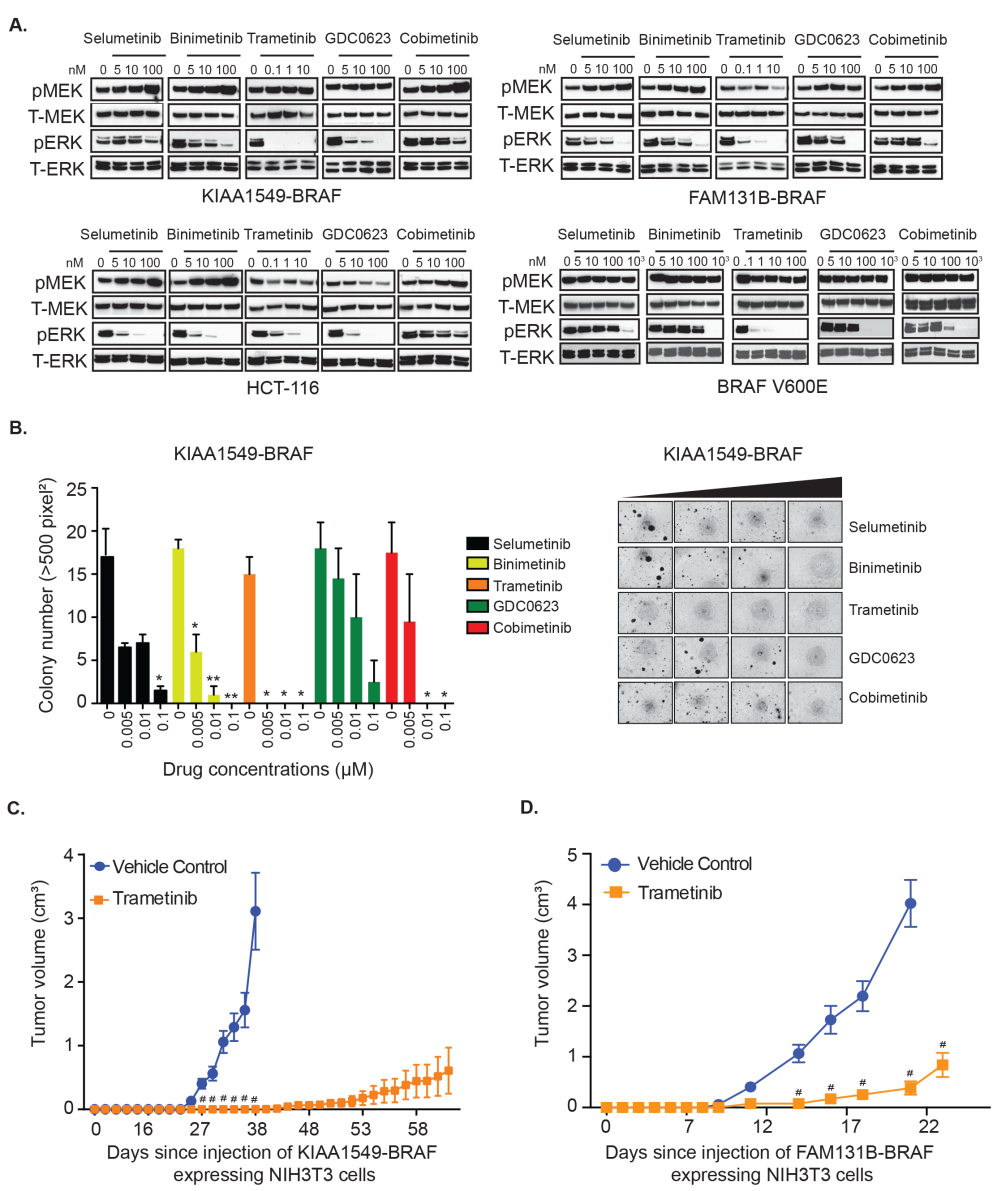
KIAA1549-BRAF and FAM131B-BRAF mediated activation of MAPK pathway and oncogenic transformation can be inhibited with MEK inhibitors **A.** Stably expressing KIAA1549-BRAF, FAM131B-BRAF, and BRAF-V600E NIH3T3 cells and a HCT-116 *RAS* mutant cancer cell line were serum starved for 24 hours and then incubated with increasing concentrations of indicated drugs for 1 hour. Corresponding cell lysates were immuno-blotted with indicated antibodies. **B.** Stably expressing KIAA1549-BRAF cells were assayed for colony formation in soft agar and anchorage independent growth in the presence of increasing concentrations of drug. Colony number quantification (left): X-axis shows increasing drug concentrations for each cell line, and Y-axis is mean colony number count (with SEM of > 3 three different images) * *p*-value < 0.05, ** *p*-value < 0.01, *** *p*-value < 0.001; image (right) is representative of one replicate of soft agar assay. **C.**, **D.** To assess *in vivo* tumor inhibition, **C.** KIAA1549-BRAF and **D.** FAM131B-BRAF NIH3T3 cell lines were injected into the flank of NSG immuno-deficient mice and treated daily with trametinib (0.33mg/kg/dose). X-axis shows days after injection and Y-axis is measured tumor volume in cm^3^ (with SEM of > 5 mice each treatment arm), # *p*-value < 0.0001 in trametinib treated *versus* vehicle control group.

To test MAPK pathway targeting against BRAF-fusions, we selected a panel of MEK inhibitors (MEKi) possessing distinct pharmacological mechanisms, namely selumetinib, binimetinib, trametinib, GDC0623 and cobimetinib, all of which had been previously shown to have activity in either *RAS*-mutant or *BRAF-V600E* mutant cell lines [25-27]. Unlike *BRAF*-altered adult malignancies or high-grade brain tumors, no PLGG patient-derived cell lines harboring BRAF-fusions have been successfully isolated or characterized. The few available PLGG patient derived cell lines, such as BT-40 with BRAF-V600E [28], a modified astrocytoma cell line [29] and Res189 with several malignant glioma mutations [30] would not serve as representative models for studying BRAF-fusion signaling and drug sensitivity. To address this, we generated heterologous cell expression systems for PLGG-associated BRAF-fusions with predictive clinical relevance [20] and assessed BRAF-fusion-driven phenotypes and biochemical activities. We evaluated MEKi in different settings- KIAA1549-BRAF fusion variants or BRAF V600E expressed in NIH3T3 cells [4], an HCT-116 *RAS*-mutant cell line, as well as a FAM131B-BRAF fusion expressing NIH3T3 cell line generated for this study. Activation levels of the MAPK pathway in response to various MEKi was evaluated *via* immunoblotting for phosphorylated-MEK (pMEK) and its downstream targets, phosphorylated-ERK1/2 (pERK1/2). We observed that trametinib, currently in clinical trials, was the most potent inhibitor, demonstrating robust, near complete abrogation of downstream pERK1/2 at concentrations lower than other MEKi [Figure 1A, Supplementary Figure 1A]. Selumetinib, binimetinib, and cobimetinib, while still inhibitory, demonstrated less robust suppression of pERK1/2 along with a dose-dependent increase in pMEK. Interestingly, we observed an inverse relationship between pMEK and pERK levels in response to selumetinib, binimetinib, and cobimetinib in the KIAA1549-BRAF, FAM131B-BRAF and HCT-116 *RAS*-mutant cells. The observation that higher inhibitor concentration induces higher pMEK levels, suggests that MAPK activation may promote feedback inhibition onto itself and suppression of MAPK signaling may relieve this inhibitory feedback [31].

Additionally, in the presence of trametinib, immunoprecipitation of MEK from drug treated cells also showed decreased interaction of myc-tagged BRAF-fusion with MEK suggesting that trametinib decreases MEK’s protein-protein interaction, thus leading to reduced activated pMEK [Supplementary Figure 1B]. Complementing the biochemical changes we observed, trametinib was also the most potent inhibitor of anchorage independent growth showing significant suppression of oncogenic colony formation [Figure 1B, *p-value < 0.05*, Supplementary Figure 1C]. In order to further demonstrate trametinib *in vivo* efficacy, we performed flank xenograft injections of NIH3T3 cells expressing KIAA1549-BRAF and FAM131B-BRAF in NSG immuno-deficient mice and treated these mice with trametinib. Mice treated with trametinib displayed prolonged suppression of tumor growth compared to vehicle-control treated mice [Figure 1C]. However, an increase in mean tumor volume was observed at later time points despite continuing treatment with trametinib, suggesting possible acquired resistance to trametinib or activation of escape pathways in tumor cells. Mice treated with trametinib did not display any signs of systemic toxicity associated with treatment.

**Figure 2:**
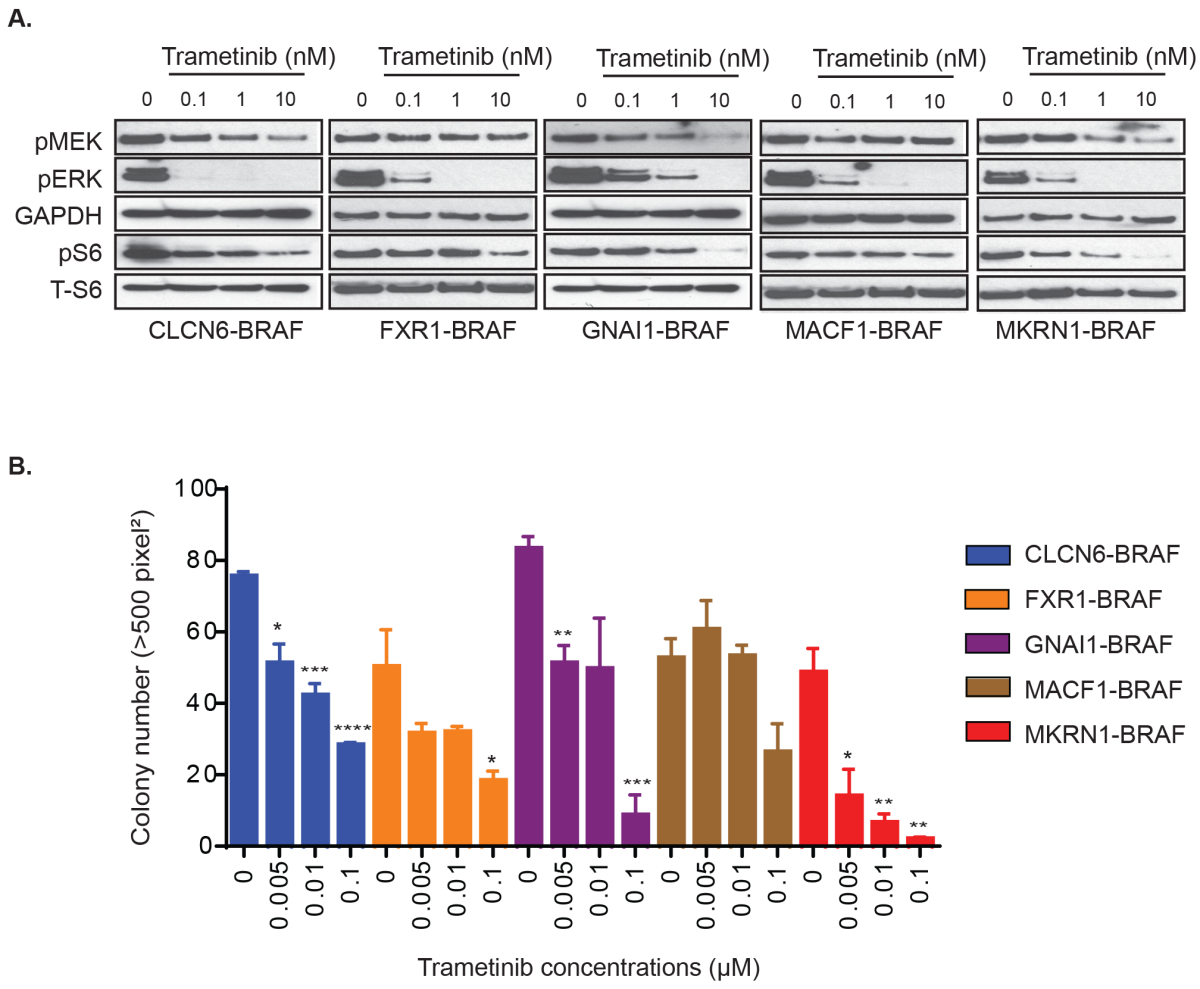
Varied effects of MEKi trametinib on various *BRAF* gene fusions **A.** GNAI1-BRAF, MACF1-BRAF, MKRN1-BRAF, FXR1-BRAF, or CLCN6-BRAF expressing NIH3T3 cells were serum starved for 24 hours and then incubated with increasing concentrations of drug for 1 hour. Corresponding cell lysates were immuno-blotted with indicated antibodies. **B.** BRAF-fusion expressing NIH3T3 were assayed for colony formation in soft agar and anchorage independent growth in the presence of increasing concentrations of drug. Colony number quantification as before (with SEM of > 3 three different images); * *p*-value < 0.05, ** *p*-value < 0.01, *** *p*-value < 0.001.

Whole genome sequencing studies have identified a number of novel BRAF-fusions in PLGGs, albeit at lower frequency than either FAM131B-BRAF or KIAA1549-BRAF fusions [9, 10]. We characterized the PLGG-associated fusions, GNAI1-BRAF, MACF1-BRAF, MKRN1-BRAF, FXR1-BRAF, and CLCN6-BRAF, by stably over-expressing in NIH3T3 (Supplementary Figure 1D) and observed activation of pERK levels by these BRAF-fusions compared to vector control (Supplementary Figure 1E). We did not observe a direct correlation between the expression level of different BRAF-fusion proteins and the level of pERK and pS6 which suggests that the aberrant kinase activity of the BRAF-fusions is not directly dependent on amount of fusion proteins available. We then assessed response to trametinib for these diverse BRAF-fusions. Each of the BRAF-fusion expressing cells exhibited dose-dependent abrogation of pERK upon trametinib treatment (Figure 2A). We also observed some suppression of phosphorylated S6 which is often used as a signaling readout for the PI3K/mTOR pathway. This correlates with previous findings in BT-40 cell line where selumetinib suppressed TORC1 signaling (mTOR component), albeit in BRAF-V600E mutation background [32]. In soft agar colony formation assay, we observed significant inhibition of colony formation with trametinib across all tested BRAF-fusions (Figure 2B, *p < 0.05 to 0.0001)*, although we required higher concentrations for suppression compared to KIAA1549-BRAF expressing cells. Interestingly, GNAI1- and MKRN1-BRAF fusions exhibited increased sensitivity and inhibition of colony formation at lower trametinib concentrations than MACF1-, FXR1-, and CLCN6-BRAF colony formation. Overall, these results suggest that while trametinib is a potent MEKi and would be effective for BRAF-fusion expressing PLGGs and adult cancers with similar BRAF-fusions, we require a deeper understanding of emergent resistance mechanisms and targeting of alternative pathways.

### Acquired resistance to MAPK pathway inhibitors in BRAF-fusion expressing cells is mediated by PI3K/AKT/mTOR pathway

**Figure 3:**
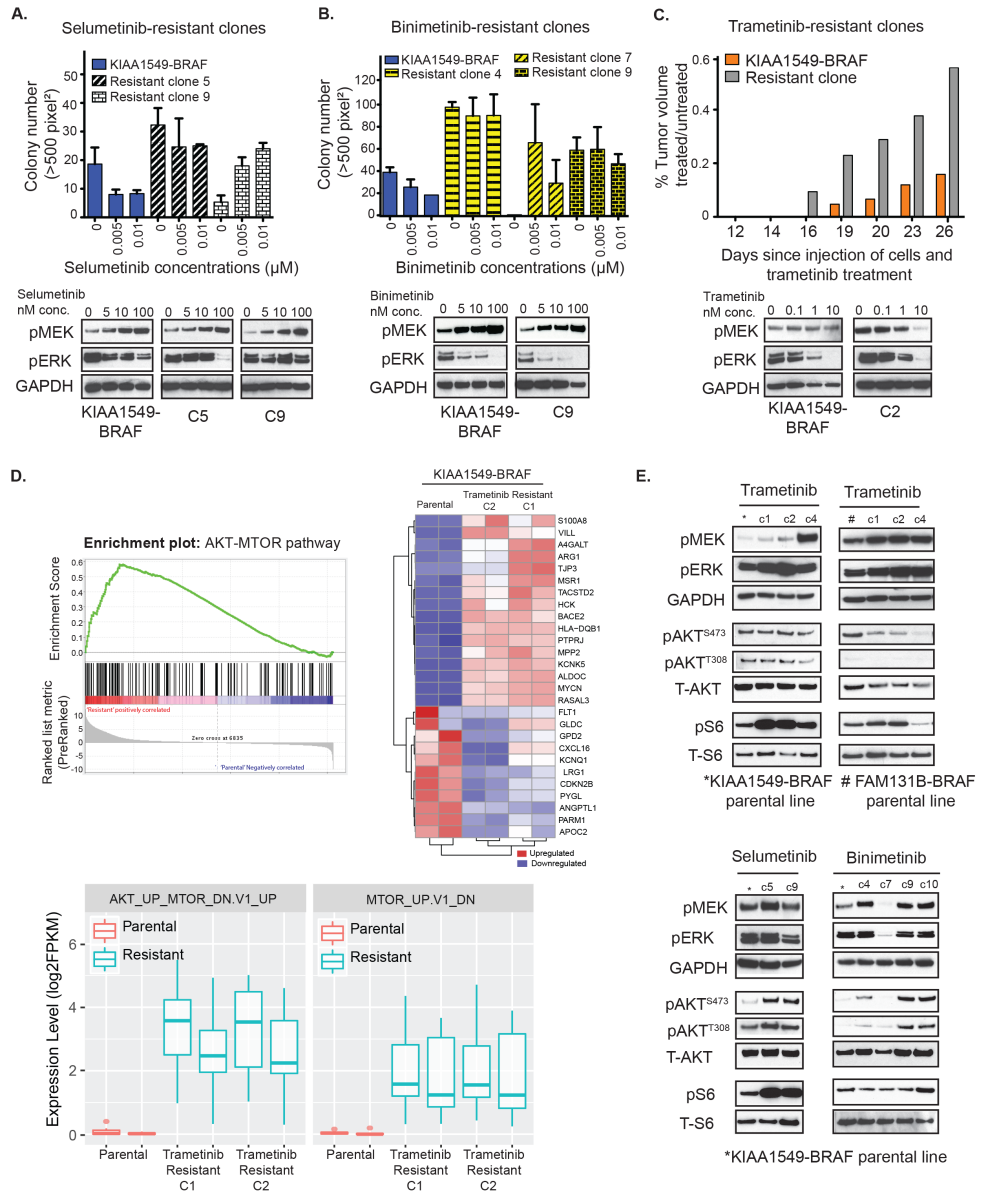
Emergent resistance to MEK inhibitors is mediated by PI3K/AKT/mTOR pathway **A.** *Top panel-* Colony formation of selumetinib-resistant KIAA1549-BRAF clones (clones 5, 9) in response to increasing drug concentrations in soft agar was quantified. X-axis shows increasing drug concentrations for each cell line, and Y-axis is mean colony number count (with SEM of > 3 three different images). *Bottom panel*- Western blot analysis of MAPK signaling pathway in clones 5 and 9 upon exposure to selumetinib. **B.** *Top panel-* Colony formation of binimetinib-resistant KIAA1549-BRAF clones (clones 4, 7, 9) in response to increasing drug concentrations in soft agar was quantified. *Bottom panel*- Western blot analysis of MAPK signaling pathway in clone 9 upon binimetinib treatment. **C.** *Top panel-* Comparison of tumor volumes in KIAA1549-BRAF expressing NIH3T3 parental cells *versus* trametinib-resistant clone (plotted bars represent mean treated tumor volume divided by mean untreated tumor volume). *Bottom panel*- Western blot analysis of MAPK signaling pathway in clone 2 upon exposure to trametinib. **D.** RNA sequencing data was analyzed using GSEA software and the top left panel shows enrichment plot for the AKT-MTOR pathway (‘AKT_UP_MTOR_DN.V1_UP’ gene set, *p*-value = 0.002) in the KIAA1549-BRAF expressing trametinib-resistant C1 and C2 clones compared to parental cells. The heat map on right shows top-ranked significantly differential gene expression in the AKT-MTOR pathway. Bottom box-plots show differential expression level of genes in 2 GSEA gene sets related to the AKT-MTOR pathways (‘AKT_UP_MTOR_DN.V1_UP’ gene set, *p*-value = 0.002 and ‘MTOR_UP.V1_DN’ gene set, *p*-value = 0.017) in the KIAA1549-BRAF expressing trametinib-resistant C1 and C2 clones compared to parental cells. Biological duplicates were used for each clone. **E.** Western blot analysis comparing MAPK and PI3K/mTOR signaling pathways in untreated, serum starved parental cell lines and corresponding MEKi resistant clones.

Clinical experience with vemurafenib and other kinase inhibitors in malignant tumors has demonstrated that acquired resistance is inevitable and can be mediated by diverse mechanisms [33-36]. As low-grade gliomas are indolent tumors and will often require months to years of treatment, thereby delaying emergent resistance to targeted therapies, it is clinically relevant to study the probable underlying resistance mechanisms and define associated pathway alterations. We sought to interrogate potential mechanisms of acquired MEKi resistance against selumetinib, binimetinib, and trametinib in KIAA1549-BRAF and FAM131B-BRAF fusions. Stably expressing BRAF-fusion cells were chronically exposed to single-agent MEKi under conditions of fusion-dependent, anchorage independent growth in soft agar. Resistant colonies that displayed continued growth and expansion in the presence of drug were isolated and called resistant clones. Interestingly, in contrast to the emergence of resistant clones with either selumetinib or binimetinib in soft agar (Figure 3A, 3B, *respectively, top graphs*), we were unable to establish trametinib resistant clones in soft agar, despite reducing the drug concentration by 10-fold. The KIAA1549-BRAF selumetinib resistant clone 5 (C5) and clone 9 (C9) displayed elevated pMEK levels upon re-exposure to selumetinib but decreasing pERK levels with higher drug concentrations (Figure 3A, bottom western blots). However, there are differences within the signaling responses of individual resistant clones as demonstrated by higher pERK suppression in selumetinib resistant clones C5 compared to C9. We observe a similar trend when the KIAA1549-BRAF binimetinib resistant clone 9 (C9) is re-e xposed to increasing drug concentrations leading to elevations in pMEK but decrease in pERK (Figure 3B, bottom western blots). This suggests similar on-target effect of selumetinib and binimetinib on MEK kinase activity in KIAA1549-BRAF parental cell lines and resistant clones. But despite this observed suppression of MAPK signaling with drug re-exposure, both selumetinib and binimetinib resistant KIAA1549-BRAF clones display anchorage independent growth in soft agar with consistent drug exposure (Supplementary Figure 2A, 2B) suggesting alternative escape pathways to single-agent MEKi therapy. We also performed statistical analysis on our soft agar data and noticed that selumetinib and binimetinib does not cause a statistically significant decrease in colony number in either of the resistant clones as compared to the parental cell line (Supplementary Figure 2C, 2D). Notably Selumetinib resistant clone 9 has a statistically significant increase in colony numbers with increasing dosage of selumetinib (*p*-value = 0.001) (Supplementary Figure 2C).

For generating trametinib resistant clones, we performed daily trametinib treatments on *in vivo* xenograft models of KIAA1549-BRAF and FAM131B-BRAF fusions and we observed consequent drug resistant flank tumor growth. We observed that the isolated trametinib-resistant clones demonstrated continued *in vivo* resistance when re-injected into the flanks of NSG mice and treated daily with trametinib (Figure 3C, *top graph*) despite showing inhibition of pERK in signaling analysis (Figure 3C, *bottom western blots*).

To assess potential resistance/escape mechanisms in our MEKi resistant clones, we performed RNA-sequencing on KIAA1549-BRAF parental and trametinib-resistant clones, C1 and C2 (as stated previously, trametinib clones were derived in mice treated with 0.33mg/kg trametinib)*.* Differential expression analyses clearly clustered resistant clones, defining a gene signature set for emergent trametinib resistance (Figure 3D, Supplementary Figure 2G). Gene set enrichment analysis (GSEA) was performed to identify altered pathways [37, 38]. Consistently, among a number of MSigDB gene sets, enrichment was observed in signatures implicating the PI3K-AKT-mTOR pathway (Supplementary Figure 2G). Confirming mTOR pathway dysregulation, gene set enrichment utilizing the “oncogenic gene set” collection, in which microarray expression data from cancer genomes have been complied, found the ‘AKT_UP_MTOR_DN.V1_UP’ gene set (*p*-value = 0.002) as significantly enriched in the trametinib resistant KIAA1549-BRAF clones C1 and C2 (Figure 3D). We found more than one AKT-MTOR pathway related gene set significantly enriched in the resistant clones (‘MTOR_UP.V1_DN’ gene set, *p*-value = 0.017) in our GSEA analysis as show by the box plot representations in Figure 3D, strongly suggesting alteration of AKT-MTOR pathway genes. We evaluated PI3K-AKT-mTOR pathway components in our BRAF-fusion clones that are resistant to trametinib, selumetinib or binimetinib *via* immunoblotting. Most MEKi resistant clones displayed a combination of either increased pAKT and/or pS6 levels (Figure 3E, Supplementary Figure 2E, F), suggesting that enhanced PI3K-AKT-mTOR pathway signaling was associated with MEKi resistance across BRAF-fusions. Previous studies have also shown that targeting MEK drives rapid reprogramming of the kinome that ultimately underlies single-agent resistance [39]. Reprogramming occurs as a result of a loss of MAPK pathway negative feedback and results in MEK inhibition, thereby inducing the expression and activation of multiple RTKs. Likewise, kinome-wide comparisons across BRAF-fusion expressing MEKi resistant clones also identified recurrent, kinome reprogramming of receptor tyrosine kinase expression in cells displaying elevated PI3K-AKT-mTOR pathway activation, suggesting a shared mechanism underlying emergent resistance (Supplementary Table 1).

**Figure 4:**
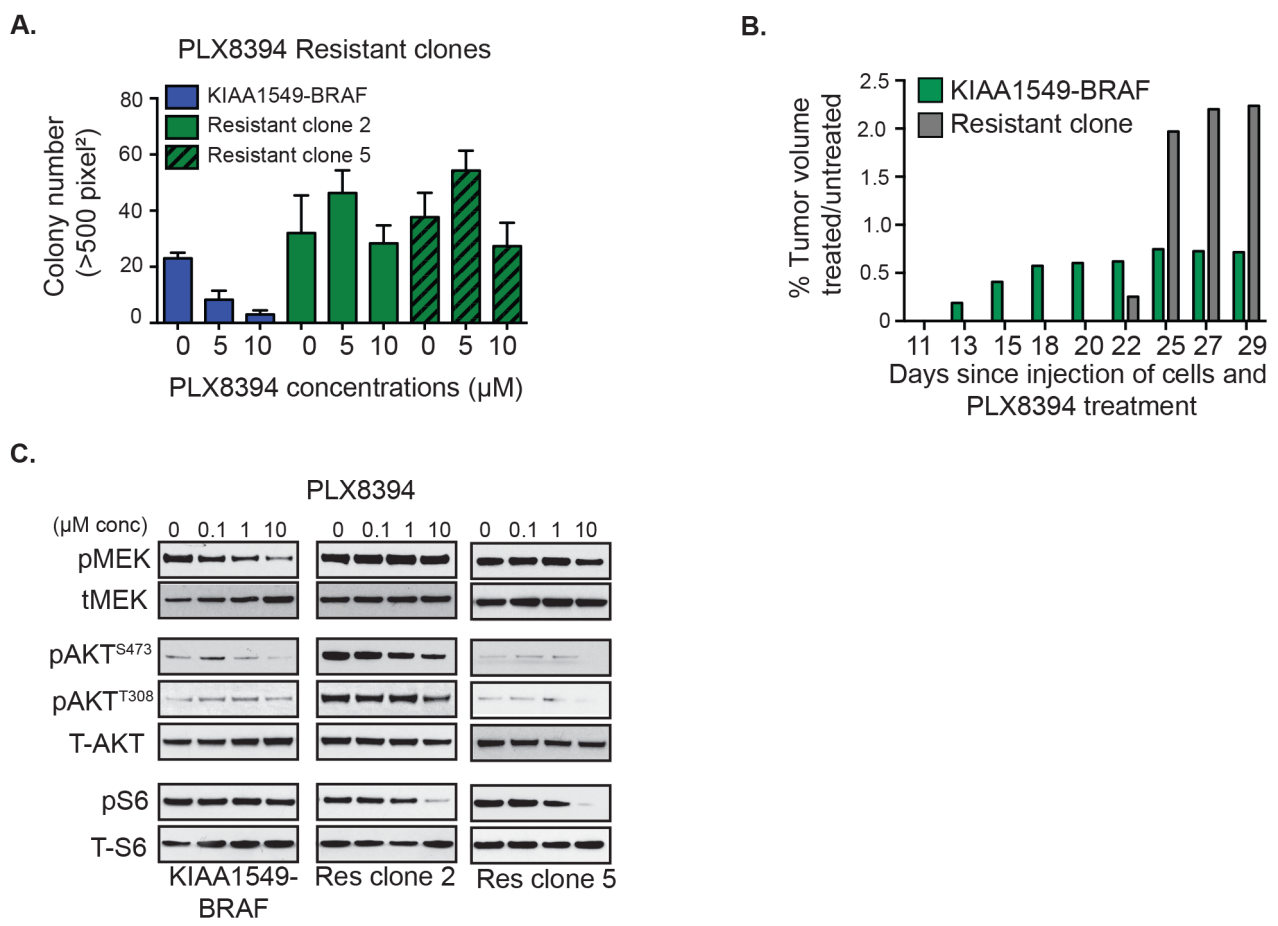
Emergent resistance to ‘paradox-breaking’ RAFi PLX8394 is mediated by PI3K/AKT/mTOR pathway activation **A.** Stably expressing *KIAA1549-BRAF* fusion cells and PLX8394 resistant cells were incubated with drug in soft agar and colony formation measured. X-axis shows increasing drug concentrations for each cell line, and Y-axis is mean colony number count (with SEM of *n* > 3). **B.** Comparisons of tumor volumes in KIAA1549-BRAF-fusion NIH3T3 parental cells *versus* PLX8394 resistant clone (graph: mean treated tumor volume divided by mean untreated tumor volume). **C.** Stably expressing KIAA1549-BRAF-fusion cells and PLX8394 resistant clones were incubated in increasing concentrations PLX8394 and lysates were immuno-blotted as indicated.

We had previously demonstrated that in contrast to vemurafenib, the second-generation RAFi “paradox breaker”, PLX8394, successfully targets BRAF-fusion [4]. We speculated whether PI3K-AKT-mTOR pathway activation could serve as a common mechanism of resistance in BRAF-fusions upon RAF targeting. We thus generated PLX8394-resistant clones for KIAA1549-BRAF expressing NIH3T3 under soft agar selection. Like MEKi-resistant clones, PLX8394i-resistant clones display *in vitro* drug-resistant colony formation (Figure 4A, Supplementary Figure 3A) as well as PLX8394-resistant *in vivo* flank-injected tumors (Figure 4B). With the linear regression statistical model, we observed no statistically significant decrease (or increase) of colony growth with increasing PLX8394 dosage in the resistant clones compared to KIAA1549-BRAF parental line (Supplementary Figure 3B). As in MEKi-resistant clones, PLX8394-resistant clones also demonstrate similar resistance trends, displaying increased pAKT (ser 473, thr 308) in comparison to parental line (Figure 4C, Supplementary Figure 3b). We also performed targeted Sanger sequencing or RNAseq variant analysis of *BRAF* and *MEK* in each of the BRAFi- and MEKi-resistant clones and did not identify any gatekeeper or other second site suppressor mutations (data not shown). Together, the data support activation of the PI3K-AKT-mTOR pathway as a recurrent resistance/escape mechanism to MAPK pathway inhibitors in the BRAF-fusion context.

### Combinatorial inhibition of MAPK and PI3K/mTOR pathways in BRAF-fusion cells prolongs suppression of tumor growth and delays acquired therapeutic resistance

**Figure 5:**
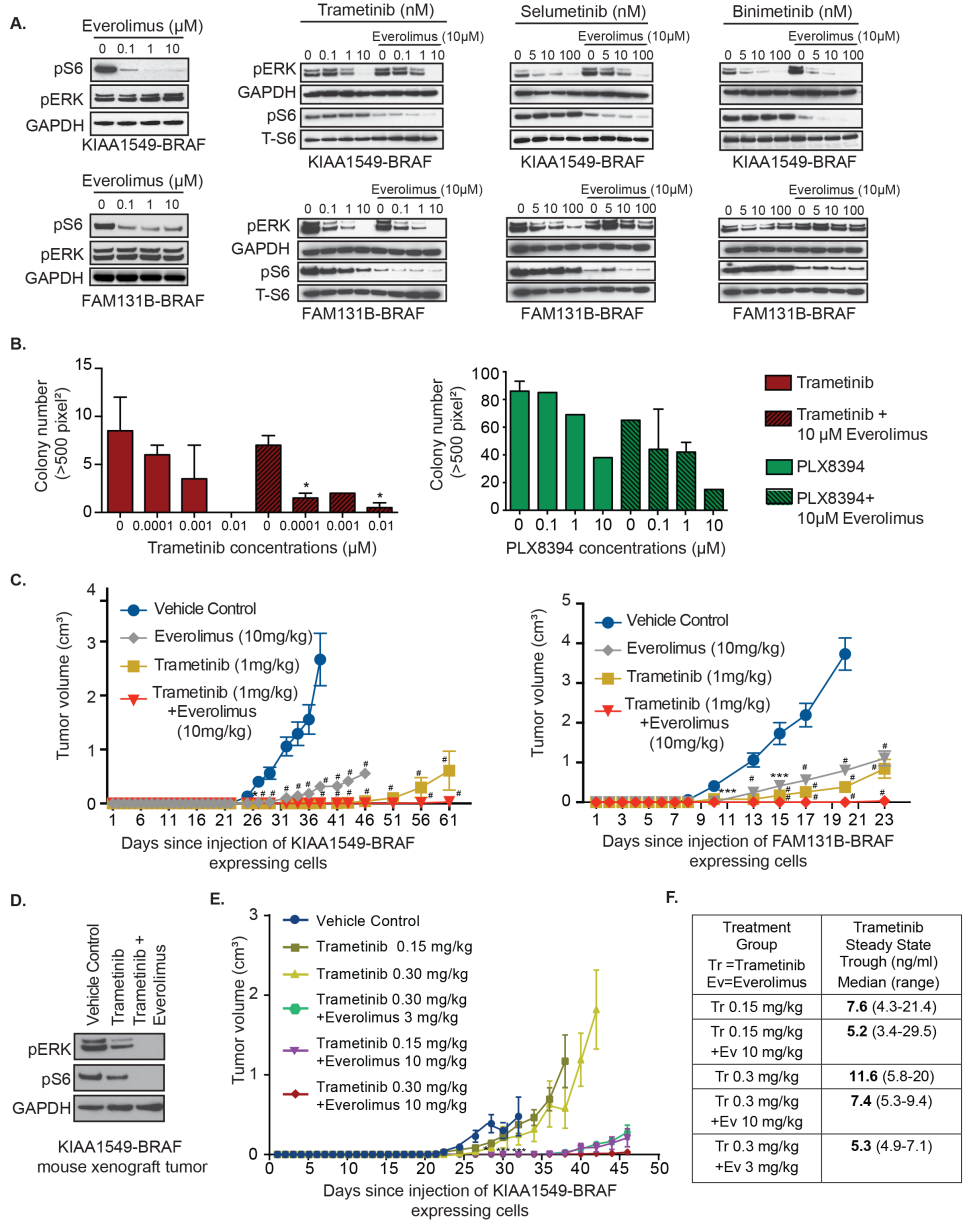
Combinatorial targeting of MEK and mTOR against BRAF-fusion displays enhanced targeting efficacy *in vitro* and *in vivo* **A.** Immunoblots of KIAA1549-BRAF expressing cell lines treated with increasing concentration of trametinib or everolimus as single agents and in combination. **B.** Anchorage independent growth of stably expressing KIAA1549-BRAF cells in the presence of increasing concentrations of trametinib as single-agent and with everolimus (*left panel*) and PLX8394 as single-agent and with everolimus (*right panel*). X-axis shows increasing drug concentrations and Y-axis is mean colony number count (with SEM of > 3 three different images), *p-value < 0.05. **C.** KIAA1549-BRAF and FAM131B-BRAF expressing cell lines were injected into the mice flanks and treated daily with trametinib, everolimus or combination of trametinib with everolimus. X-axis shows days after injection and Y-axis is measured tumor volume in cm^3^ (with standard error mean). **D.** Immuoblot of KIAA1549-BRAF mouse tumor lysates from panel D assessing on-target effect of inhibitors. **E.** KIAA1549-BRAF expressing cell lines were injected as flank xenografts and mice treated daily with lower doses of trametinib and everolimus as indicated (*n* = 10, SEM values shown). For mouse experiments, * *p*-value < 0.05, ** *p*-value < 0.01, *** *p*-value < 0.001 and ^#^ *p*-value < 0.0001 between vehicle control and respective treatment group.

As PI3K-AKT-mTOR pathway is the common emergent mechanism of RAFi- and MEKi-resistance in BRAF-fusion expressing cells, we hypothesized that co-targeting of both pathways could display enhanced therapeutic efficacy. When incubated with everolimus as single-agent mTORi, KIAA1549-BRAF variants expressed in NIH3T3 displayed varying levels of decrease in pS6 and no effect on pERK (Figure 5A, *left panel*, Supplementary Figure S4A). However, in soft agar assays, single-agent everolimus did not suppress KIAA1549-BRAF driven colony formation (Supplementary Figure 4B). In Figure 5A, we noticed incomplete suppression of pS6 levels with 0.1 µM everolimus on KIAA1549-BRAF expressing cells but in FAM131B-BRAF cells, 10 µM of everolimus only partially suppressed pS6. For the combinatorial assay, we tested 10 µM everolimus as doses higher than that would not be clinically achievable. Interestingly, the addition of everolimus with trametinib, selumetinib, or binimetinib further decreased pS6 signal in both KIAA1549-BRAF and FAM131B-BRAF expressing NIH3T3 (Figure 5A, *respectively,* Supplementary Figure 4C). In FAM131B-BRAF cells, we see an unexpected effect of everolimus on pERK levels when combined with selumetinib and binimetinib as pERK levels are higher compared to MEKi alone treatment (Figure 5A). We hypothesize that this could be due to feedback response of the MAPK pathway when mTORC is targeted downstream and will explore this in future experiments. Combining PLX8394 with everolimus caused reduction in pS6 signal but had minimal effect on pMEK (Supplementary Figure 4D). In soft agar phenotypic assays, combinatorial targeting with either PLX8394 or MEKi and everolimus resulted in suppression of BRAF-fusion driven colony growth (Figure 5B, *p*-value < 0.05, Supplementary Figure 4E, 4F) with better suppression using trametinib and everolimus combination. Similar effect of trametinib and everolimus co-treatment was observed *in vivo* with near total inhibition of KIAA1549-BRAF or FAM131B-BRAF driven tumor growth (Figure 5C, *left and right graph respectively*). We also observed on-target effect of the inhibitors on signaling pathways in treated mice tumors as seen by decreased pERK in trametinib-treated mice and both pERK and pS6 in combo-treated mice (Figure 5D). We did not observe any adverse effects of combined treatments in mice.

Prior early phase clinical studies in adults have demonstrated dose-related toxicities when combining trametinib and everolimus [40]. Therefore we scaled our mouse treatment doses to therapeutically relevant human doses, we assessed the lowest level of drug dosage that could result in sustained suppression of BRAF-fusion driven tumor growth *in vivo*. We used KIAA1549-BRAF expressing cells in our flank xenograft model and decreased the dosage of trametinib as single-agent to 0.15 mg/kg and 0.3mg/kg and, in combination with everolimus at 0.3 mg/kg and 10 mg/kg. We observed similar levels of prolonged inhibition of BRAF-fusion driven tumor growth with 0.3mg/kg trametinib combined with 10mg/kg everolimus compared to 1mg/kg trametinib and 10mg/kg everolimus suggesting effectiveness of lower trametinib doses (Figure 5E). We also noticed that combining either 0.3 or 0.15 mg/kg of trametinib with 3 or 10mg/kg everolimus was still effective at maintaining prolonged suppression of tumor growth as compared to lower doses of single-agent trametinib treatment (Figure 5E, Supplementary Figure 4G). Our pharmacokinetic analysis of the steady state trough concentrations of trametinib measured in mice treated with trametinib oral gavage (0.15 or 0.3 mg/kg) as a single agent or with everolimus (3 or 10 mg/kg) were variable. The trough concentration increased in proportion to dose in mice treated with trametinib alone, however, dose proportionality of trametinib was not apparent in mice treated with trametinib in combination with everolimus (Figure 5F). These findings suggest that lower combinatorial doses of trametinib and everolimus would be effective in targeting BRAF-fusion driven oncogenesis and warrant further clinical testing.

**Figure 6:**
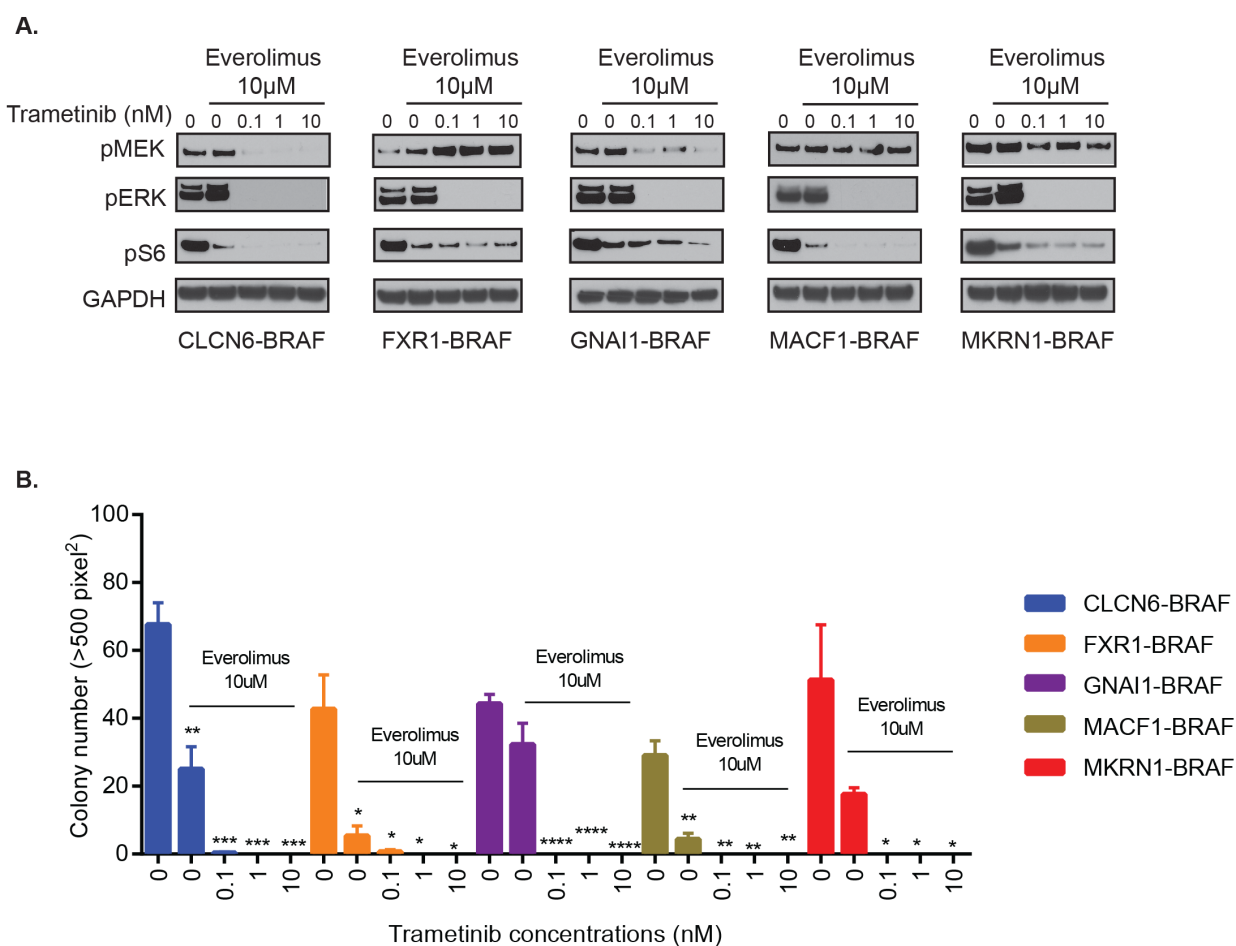
Combinatorial treatment of additional BRAF-fusions with MEKi and mTORi shows inhibition of signaling and oncogenic growth **A.** GNAI1-BRAF, MACF1-BRAF, MKRN1-BRAF, FXR1-BRAF or CLCN6-BRAF expressing NIH3T3 cells were exposed to indicated inhibitors for 1 hour and subjected to western blot analysis. **B.** Stably expressing BRAF-fusion lines were assayed for colony formation in soft agar in the presence of increasing concentrations of trametinib and 10µM everolimus. Colony number quantification (left): X-axis shows increasing drug concentrations for each cell line, and Y-axis is mean colony number count (with SEM of > 3 three different images), *p-value < 0.05 to 0.0001.

Next, we assessed the effect of MEKi and mTORi on additional BRAF-fusions, GNAI1-BRAF, MACF1-BRAF, MKRN1-BRAF, FXR1-BRAF, and CLCN6-BRAF. We found potent suppression of pERK and pS6 signals (Figure 6A) and corresponding decrease in BRAF-fusion driven colony formation in soft agar with trametinib and everolimus co-treatment (Figure 6B, *p-value < 0.05 to 0.0001*). Thus, MEK and mTOR co-targeting may represent a successful, generalized targeting strategy against diverse BRAF-fusions that warrants careful testing in PLGGs as well as other pediatric and adult cancers where additional BRAF-fusions are being discovered.

## DISCUSSION

In our present study, we describe sensitivity to targeted inhibitors and resistance/escape mechanisms driven by BRAF-fusions that are prevalent in pediatric low-grade gliomas and found in several adult cancers. We have previously demonstrated that the most common BRAF-fusion in PLGGs, KIAA1549-BRAF, has innate resistance to first-generation RAFi vemurafenib (research analog PLX4720), and instead is paradoxically activated upon PLX4720 treatment resulting in accelerated tumor growth [4]. Such studies have proven predictive of PLGG patient responses to currently available BRAFi [20], and highlight the need for robust mutation-specific targeting of BRAF-fusion driven MAPK signaling pathway. Our current findings with KIAA1549-BRAF and FAM131B-BRAF show robust responsiveness to clinically available MEKi such as trametinib. Here, we also validate potency of MEKi treatment against multiple, previously uncharacterized BRAF-fusions that have been discovered in PLGGs, some of which, like MKRN1-BRAF, co-occur in diverse adult cancers [12]. We further demonstrate that acquired resistance to MEKi or the previously studied PLX8394 (‘paradox-breaker’ RAFi) [4] eventually develops in BRAF-fusion expressing heterologous models, with the PI3K-AKT-mTOR signaling cascade being the major escape mechanism for BRAF-fusions. To target such emergent resistance to single-agent therapy, we demonstrate efficacy of combinatorial targeting using MEKi and mTORi for BRAF-fusion driven tumors.

Numerous studies, largely in melanoma, have demonstrated that successful targeting of the MAPK signaling cascade is dependent on discrete, mutation-specific response to inhibitors among wild-type *BRAF, BRAF-V600E* and mutant-*RAS* tumors [16-18, 41] This is partly due to altered protein-protein interactions, RAF-heterodimerization, and kinase-substrate interactions as well as reorganization of a highly dynamic signaling network in response to targeted inhibitors [42]. RAF-targeted therapies such as vemurafenib and dabrafenib are approved treatments for malignant melanomas with BRAF-V600E/K mutations. However, due to emergent resistance to single-agent RAFi in patients [43, 44], combination treatments of BRAFi (dabrafenib) with MEKi (trametinib) have been developed for melanomas [45] and are also being tested in pediatric patients with plexiform NF-1, BRAF-V600E refractory/relapsed tumors (NCT02124772). Similar selectivity and efficacy of trametinib was also reported as a single-agent in wild-type *BRAF/NRAS*, *NF1*-altered melanomas [46]. However, recent studies have highlighted important mechanistic differences in sensitivity to different MEK inhibitors based on mechanism of inhibition, mutational context, and induction of RAF-MEK complexes as seen in mutant-*RAS* tumors [42, 47].

In contrast to *BRAF* point mutations, *BRAF* gene fusions define a distinct mechanism of BRAF activation in several solid tumors across adult and pediatric patients [12, 48]. In our model systems, we show successful targeting of BRAF-fusion driven MAPK signaling with different MEKi, including selumetinib, binimetinib, trametinib, cobimetinib and GDC0623. Distinct mechanisms of inhibition previously observed for cobimetinib and GDC0623 in RAS-mutant cells [27] may in part parallel their inhibition profile against BRAF-fusions as seen in our study. Selumetinib is currently undergoing clinical testing for recurrent/refractory PLGGs where patients harbor KIAA1549-BRAF or BRAF-V600E or both and phase I data has shown 8/38 sustained partial responses (NCT01089101). However, differences in MEKi sensitivity among BRAF-fusion, RAS-mutant or BRAF-V600E mutations as well as induced MEK phosphorylation observed in our study further highlight the unique signaling context invoked by the BRAF-fusion. We also noticed that induction of MEK phosphorylation by colmetinib, selumetinib, and blinmetinib correlated with lower efficacy of BRAF-fusion targeting, whereas trametinib, an allosteric inhibitors that also blocks feedback-mediated MEK phosphorylation, was more potent and may provide greater promise in the PLGG clinical setting. Our preclinical study has shown trametinib as the most potent MEKi against mutually exclusive, novel BRAF-fusions expressed in heterologous model systems, strongly supporting further clinical testing. In line with our conclusion, *PAPSS1-BRAF*, a novel fusion found in previously described “pan-negative” melanoma, has also been shown to be sensitive to trametinib [23] and good clinical outcome with trametinib has been reported in a melanoma patient harboring a rare *ZKSCAN1-BRAF* fusion [12].

Several clinical studies evaluating single-agent BRAFi or MEKi in melanomas have shown that the MAPK pathway can be remarkably plastic and drug resistance nearly inevitable, requiring additional strategies to delay or prevent resistance. Current strategies for BRAF-V600E melanomas that delay, but have yet to eliminate acquired resistance to BRAFi include combinatorial therapy with BRAFi and MEKi, co-targeting of the MAPK and PI3K pathways [49, 50], and the combination of small molecule inhibitors with immunotherapy [51]. Whether single-agent resistance to targeted inhibitors will occur in *BRAF*-mutant PLGGs remains unknown, clinical observation with chemotherapy suggests that repeated chemotherapy treatment for progressive PLGG produces similar outcomes as initial treatment [52]. However, we demonstrate that the BRAF-fusion signaling network in our model systems is susceptible to dynamic responses to targeted inhibitors. Our *in vitro* and *in vivo* studies show that PLGG-associated KIAA1549-BRAF and FAM131B-BRAF fusions utilize the PI3K-AKT-mTOR pathway as an escape mechanism in response to BRAF/MEK inhibition. Since the KIAA1549-BRAF fusion is also found in adult cancers such as breast cancer and sarcomas [12], our findings could inform emergent drug resistance in multiple cancers harboring BRAF-fusions.

Prior studies showing activation of the mTOR pathway in PLGGs [53-55] combined with our current findings in drug resistant BRAF-fusion cell lines indicate that mTOR may be an ideal target for combinatorial therapy along with MEKi. Additionally, PI3K pathway activation has been suggested to be associated with a more aggressive subset of PLGGs [56]. As a single-agent therapy for PLGGs, everolimus has recently been shown to have limited clinical efficacy, though molecular profiling of underlying mutations was not performed [57]. An additional on-going clinical trial for adult recurrent/progressive low-grade glioma is further defining differential everolimus responsiveness based on tumor associated S6 phosphorylation (NCT00831324). Everolimus is relatively well tolerated with long-term treatment as has been shown in the EXIST-1 study (NCT00789828), where everolimus was used to treat tuberous sclerosis patients (germ-line mutations in TSC1/2) with subependymal giant cell astrocytomas (SEGAs) [58]. Our current work provides preclinical rationale for combinatorial targeting of MAPK and mTOR pathways with trametinib and everolimus in BRAF-fusion bearing PLGGs. This correlates with our previous study where combination of everolimus with selumetinib was found efficacious in targeting the BT-40 (BRAF-V600E) astrocytoma cell line as well as NIH3T3 expressing KIAA1549-BRAF [21]. However, a recent clinical study failed to identify tolerable drug dosage of combination trametinib and everolimus in adult patients with advanced solid tumors, raising questions about the safety and tolerability of this drug combination [40]. While this study used an adult patient cohort with potentially high mutational burden and previous exposure to chemotherapy and/or other treatments, our *in vivo* study found that lower combinatorial drug doses were efficacious and non-toxic in treatment-naïve BRAF-fusion xenograft mice with low mutational burden. These findings suggest that combinatorial targeting of MAPK and PI3K/mTOR pathways against mutually exclusive mutational events in PLGGs such as BRAF-fusions should be further explored in the pediatric setting. Furthermore, the effectiveness of trametinib and everolimus co-targeting against multiple distinct BRAF-fusions to in our study suggests that such strategies may offer synergistic targeting opportunities in other tumor subtypes, including melanoma where BRAF-fusions have begun to be characterized [23, 48].

## MATERIALS AND METHODS

### Vector construction and generation of stable cell lines

Generation of KIAA1549-BRAF constructs and stably expressing NIH3T3 lines were previously described [4]. Briefly, BRAF stably expressing cell lines were produced using Platinum E packaging lines with a gateway-modified pMX-retroviral per manufacturer’s suggestion (Cell Bio Labs). NIH3T3 cells (obtained from ATCC in 2009 and maintained according to protocol) were infected with retrovirus in accordance with the manufacturer’s suggested protocol and then selected for stable transgene expression after 2-4 weeks of selection in puromycin. The same strategies were used for the generation of FAM131B-BRAF, MACF1-BRAF, FXR1-BRAF, MKRN130-BRAF, CLCN6-BRAF, and GNAI1-BRAF constructs/lines based on published description or independent confirmation of their genomic alterations in patient-derived samples [9, 10]. Expression was validated by western blotting for Myc-tagged BRAF-fusions (Invitrogen R951-25, 1:5000) as well as *via* RNA sequencing as part of expression profiling studies. All cells were routinely tested for mycoplasma infection.

### Cellular transformation assays

The ability of the BRAF mutants to transform cells was assessed by anchorage independent growth, as determined in soft agar assay (Cell Biolabs^®^ San Diego, CA) and quantified as previously described [7]. We plated 4x10^5^ cells for the respective soft agar assays.

### Statistical analysis

*P*-values were calculated using *t*-tests (correction for multiple comparisons using the Holm-Sidak method). For soft agar assay with drug resistant clones, linear regression was performed for each cell line (i.e. parental and individual resistant clones) and each drug to determine statistically significant association between dose and value. This data was additionally visualized on a per drug basis using box-plots with a line displaying the linear trend. Statistical analysis and visualizations were generated using the R framework and ‘ggplot2’ package.

### Western blot analysis

Protein concentrations of cell samples were determined by using Pierce 660nm Protein Assay and run on NuPAGE precast gels (4%-12% Bis-Tris or Tris-acetate). Immunoblots were incubated with the indicated primary antibodies per manufactures’ recommendations and as described previously [4]. For MAPK and PI3K/mTOR pathway analysis, pMEK (#9154), MEK (#4694), pERK (#4370), ERK (#4695), pAKT Ser473 (#4060), pAKT Thr308 (#4056), AKT (#2920), pS6 (#2215) and S6 (#2317) antibodies from Cell Signaling were used. HRP-conjugated Beta-Actin, and HRP-conjugated GAPDH were also obtained from Cell Signaling Biotechnology, Inc.; anti-ERK 1/2 was obtained from Promega; anti-BRAF was obtained from Abcam.

### Cell-based drug studies

Cells were plated at 1x10^6^cells/ml in presence of serum and then serum starved for 24 hours. Cells were exposed to indicated concentrations of drug for 1 hour and then lysed with RIPA lysis buffer (50 mM Tris, 150 mM NaCl, 0.1% SDS, 0.5% sodium deoxycholate, 1% Nonidet P-40, Roche complete protease inhibitor mixture tablets, and 100x Pierce phosphatase inhibitor mixture) while being maintained at 4°C. Cell suspension was centrifuged and clarified lysates isolated. Lysates were protein quantified and diluted to a standard concentration with 2X LDS buffer so all aliquots had the same protein quantification prior to western blotting. Trametinib was provided by GlaxoSmithKline. PLX8394 was provided by Plexxikon. Selumetinib, binimetinib, GDC0623, and cobimetinib were purchased from Selleck Chemicals (Selleckchem.com). Drug studies in soft agar assays were performed as described previously [4].

### Co-immunoprecipitation assays

Cells were plated in 10cm plates at 1x10^6^cells/ml in presence of serum for 24 hours and then serum starved for 24 hours. Cells were incubated with indicated (and concentrations) for one hour and lysed in 600 µL buffer containing 0.5% NP40, 20 mM Tris 7.5pH, 137 mM NaCl, 10% Glycerol, 1mM EDTA, and protease/phosphatase inhibitor. Cell lysates were rotated at 4 deg C for 15 minutes and centrifuged at 16,000 RCF for 20 minutes. The supernatant was isolated, protein concentration quantified, and 100 µL of was placed in an eppendorf with 50µL/sample of a prepared mixture of coupled Invitrogen A Dynabeads- MEK-1 antibody (Millipore) 8µg/8µl, and rotated at 4°C for 2 hours. Beads were washed 3 times in 1mL of lysis buffer and 1xPBS. Beads were resuspended in 80 µL of 2x LDS and then immunoblotted as described above.

### Generation of drug resistant clones

PLX8394-, selumetinib- and binimetinib-resistant BRAF-fusion clones were generated by chronic exposure in soft agar. KIAA1549-BRAF, KIAA1549-BRAF-fusion-3, and FAM131B-BRAF NIH3T3 cells were plated in soft agar at 1x10^6^ cells/ml density (Cell Biolabs CytoSelect 96-well cell transformation assay CBA-135). Cell and top layers were prepared at a final concentration of 0, 0.005 and 0.01 µM of selumetinib and binimetinib, and at 0, 1 and 10 µM of PLX8394. Fresh media with drug was replenished weekly until resistant cell colony expansion was observed and cells were recovered. Colonies were processed per manufacturer’s protocol and further expanded. Prolonged treatment of soft agar cultures with trametinib failed to elicit resistant clones and trametinib resistant clones were generated *in vivo* in flank tumor models. NSG mice were flank injected with 1x10^5^ cells of KIAA-BRAF and FAM131B-BRAF and treated daily with trametinib (1 mg/kg). Animals that displayed resistant flank tumors were euthanized per approved IACUC protocol and tumor samples were recovered and isolated cells expanded in culture under continued drug exposure.

### Animal drug studies

The Children’s Hospital of Philadelphia Institutional Animal Care and Use Committee approved all animal protocols. Homozygous NSG strain immunodeficient mice were bred in our animal facility and housed under aseptic conditions. Trametinib inhibition studies were performed in a xenograft mouse model by pre-treating with daily oral gavage (1 or lower doses mg/kg/dose) for one week prior to injecting the fusion-harboring cell lines subcutaneously into the flanks of NSG immunodeficient mice. For testing everolimus as a single agent and in combination, the mTORi was administered *via* oral gavage (3 or 10mg/kg dose). For the drug dosage study, we used indicated concentration of inhibitors in combination. PLX8394 inhibition studies were performed using drug containing chow (1600 mg per 1 kg of chow) provided by Research Diets/Plexxikon, also pre-treating the animals for one week prior to injection and continuing on treatment until endpoints were met. For each drug trial, there were on average 10 mice per treatment arm. Tumor growth was measured with calipers on a 3-4x/weekly basis. Ellipsoid tumor volume was calculated using the formula: volume = ½*L*W^2^.

### Quantification of trametinib in plasma (pharmacokinetic analysis)

Mouse plasma samples were spiked with an isotopically labelled internal standard (Trametinib-d4; Santa Cruz Biotechnology, Dallas, TX) and trametinib was extracted by liquid-liquid extraction using acetonitrile. Extracts were injected onto an Xbridge C18 column (50 x 2.1 mm, 2.5µm; Waters Corporation, Milford, MA), maintained at 40 °C and eluted with a binary mobile phase gradient using 0.1% formic acid in water (A) and 0.1% formic acid in acetonitrile (B) with a constant flow rate of 0.3 ml/min. The initial mobile phase condition of 50:50, A:B, was held until 0.1 min, when the composition changed to 5:95, A:B gradually until 1.0 min. From 1.0 min to 1.3 min, the mobile phase held at 5:95, A: B, then reverted back to the initial conditions (50:50, A:B) at 1.31 min. Positive ion MS/MS with multiple reaction monitoring (m/z 616 to m/z 491 for trametinib) was used for analyte detection. The lower limit of quantification (LLQ) for trametinib was 0.125 ng/ml for a 15µl aliquot of mouse plasma with a higher limit of quantification (HLQ) of 500 ng/ml.

### Targeted Sequencing of MAPK inhibitor resistant clones

Sanger sequencing was performed on PCR-amplified products encompassing the MEK and C-terminus BRAF kinase domain on all PLX8394, trametinib, binimetinib, and selumetinib resistant clones.

### RNA sequencing analysis

RNA extraction was performed per manufacturer’s protocol (Promega SV Total RNA Isolation System). For sequencing, mRNA was fragmented into ∼300bp short fragments, libraries were prepared using the TruSeq Stranded mRNA Sample Prep Kit and 100bp pair-end sequencing performed on an Illumina HiSeq 2500. Reads were mapped to UCSC mouse genome reference mm10 using STAR along with UCSC mm10 gene annotation GTF file. Read counts for each gene was calculated using HTSeq-count with the following settings: —mode intersection-strict —stranded yes —minaqual 10 —type exon —idattr gene_id -r name. Based on the HTSeq-count output, gene expression normalization as well as differential expression was calculated using the DESeq2 package [59]. Gene Set Enrichment Analysis (GSEA) was performed using C2 (curated gene sets) and C6 (oncogenic signatures) [37, 38].

## SUPPLEMENTARY MATERIALS FIGURES AND TABLE


