# Supplementary material for: Overcoming resistance to single-agent therapy for oncogenic BRAF gene fusions via combinatorial targeting of MAPK and PI3K/mTOR signaling pathways
Source: Oncotarget. 2017 Sep 15;8(49):84697–713. doi: 10.18632/oncotarget.20949 (PMC5689567; doi:10.18632/oncotarget.20949)
Supplement: Supplementary file 1 [file oncotarget-08-84697-s001.pdf]

Overcoming resistance to single-agent therapy for oncogenic *BRAF* gene fusions *via* combinatorial targeting of MAPK and PI3K/mTOR signaling pathways

SUPPLEMENTARY MATERIALS

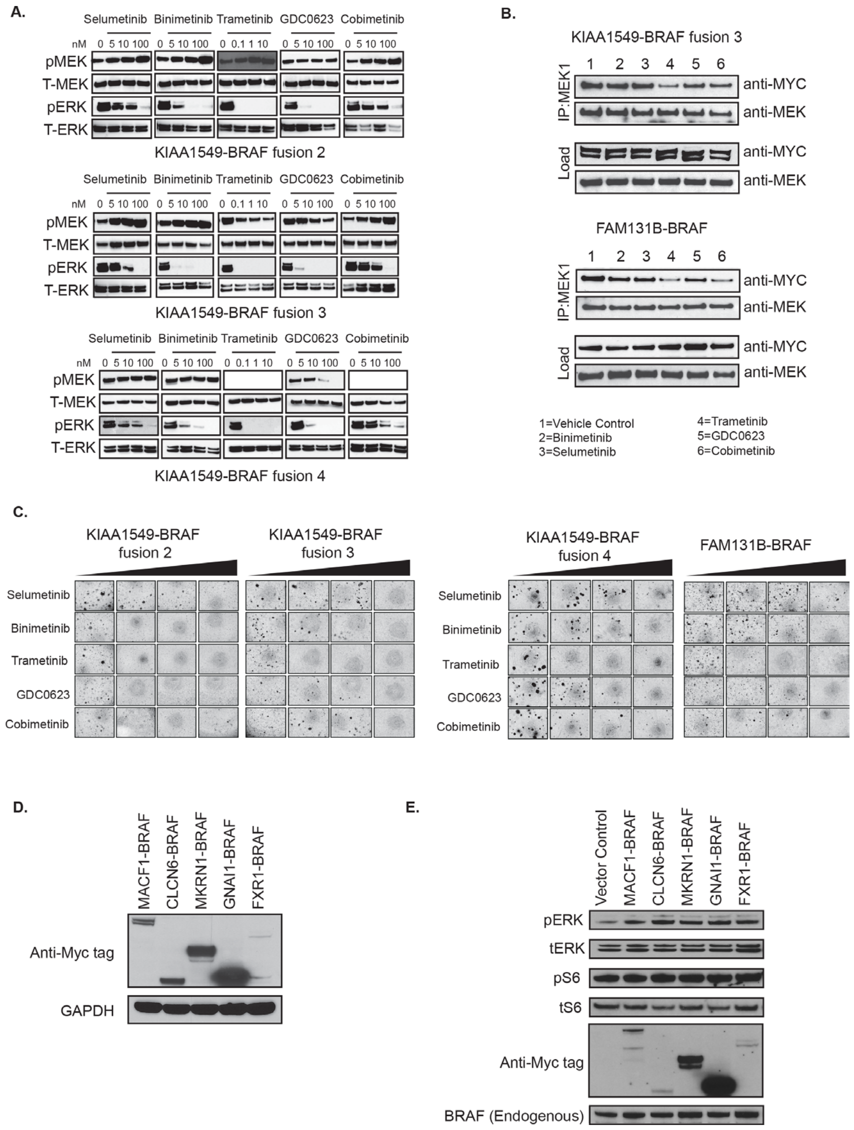

Supplementary Figure 1: Additional KIAA1549-BRAF fusions display consistent inhibition with trametinib disrupting BRAF-fusion/MEK interactions. Previously characterized KIAA1549-BRAF fusions are used as described [4], fusion-2 is the short-form of the KIAA1549-BRAF fusion found in human tumors, fusion-3 and fusion-4 are KIAA1549-BRAF fusion proteins further truncated to enhance expression and eliminate putative KIAA1549 transmembrane domains with fusion-3 further modified to include an N-terminus Src myristoylation sequence for membrane localization. (A) Indicated cell lines were treated with MEKi and lysates probed for phospho-MEK (pMEK), total MEK (T-MEK), phospho-ERK (pERK), and total ERK (T-ERK). (B) Immunoprecipitation of endogenous MEK from BRAF-fusion expressing cells after exposure to MEKi. Both beads and lysates were probed for anti-MYC (representing Myc-tagged BRAF-fusion) and anti-MEK. (C) Colony formation assays in the presence of increasing concentrations of indicated drug. Images are representative results. (D) Expression level of GNAI1-BRAF, MACF1-BRAF, MKRN1-BRAF, FXR1-BRAF, and CLCN6-BRAF in stably expressing NIH3T3 cells.

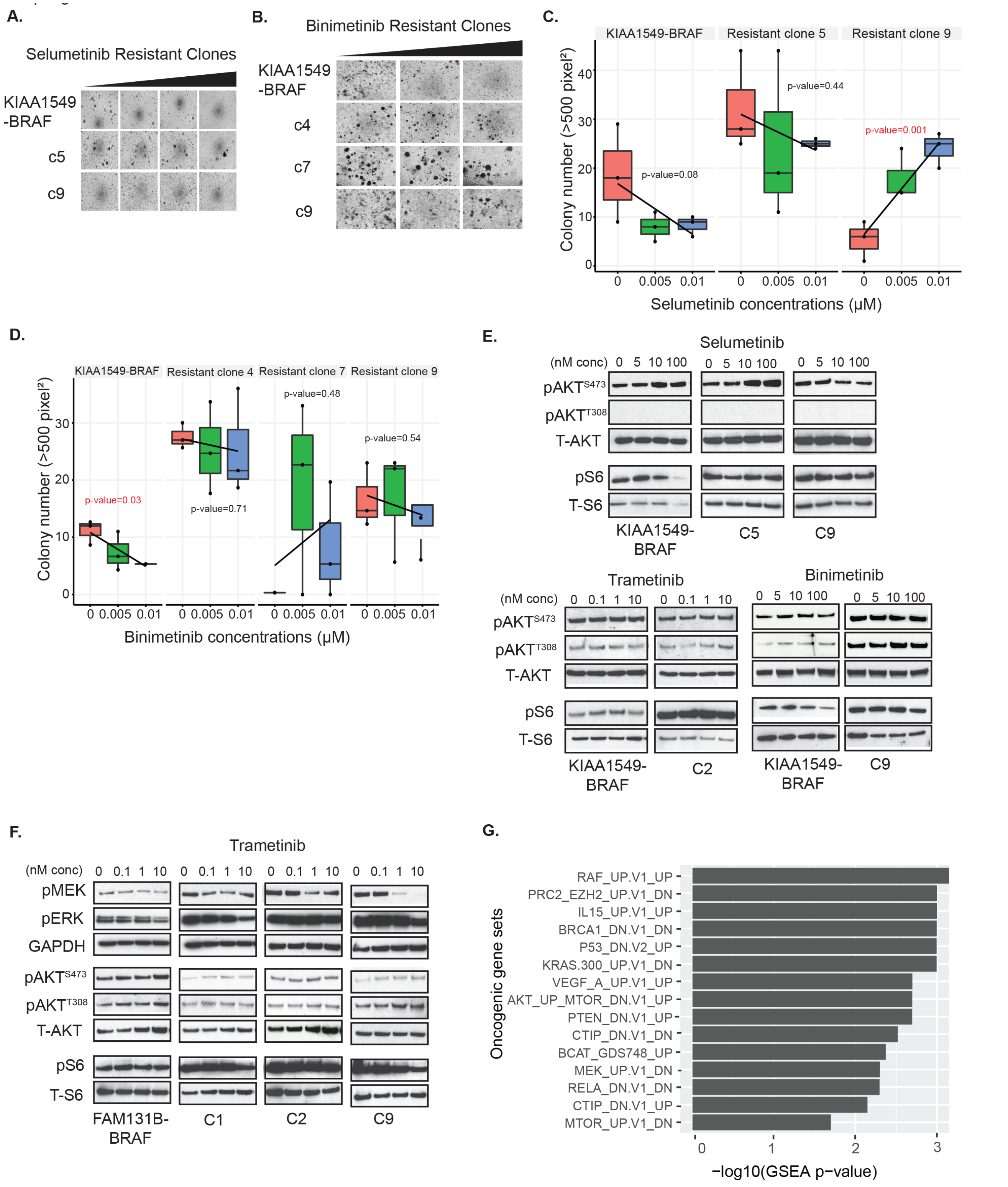

Supplementary Figure 2: MEKi resistant BRAF-fusion clones demonstrate persistent activation of the PI3K/mTOR pathway when re-exposed to MEKi. (A, B) Representative soft agar images of (A) selumetinib and (B) binimetinib resistant clones used in the analysis shown in figure 3A and 3B respectively. (C, D) Linear regression analysis plots for colony numbers shown in figure 3A and 3B respectively. (E) KIAA1549-BRAF and (F) FAM131B-BRAF expressing NIH3T3 parental cells and respective MEKi resistant clones generated *in vitro* (selumetinib, binimetinib) or *in vivo* (trametinib) were incubated with increasing concentrations of the specified inhibitor. Western blot analysis of both MAPK and PI3K/mTOR signaling pathways was performed. (G) GSEA pathway analysis of RNAseq data comparing significantly enriched pathways in trametinib resistant cells to KIAA1549-BRAF expressing parental cells- plot shows significant pathways upregulated in MEKi resistant clones.

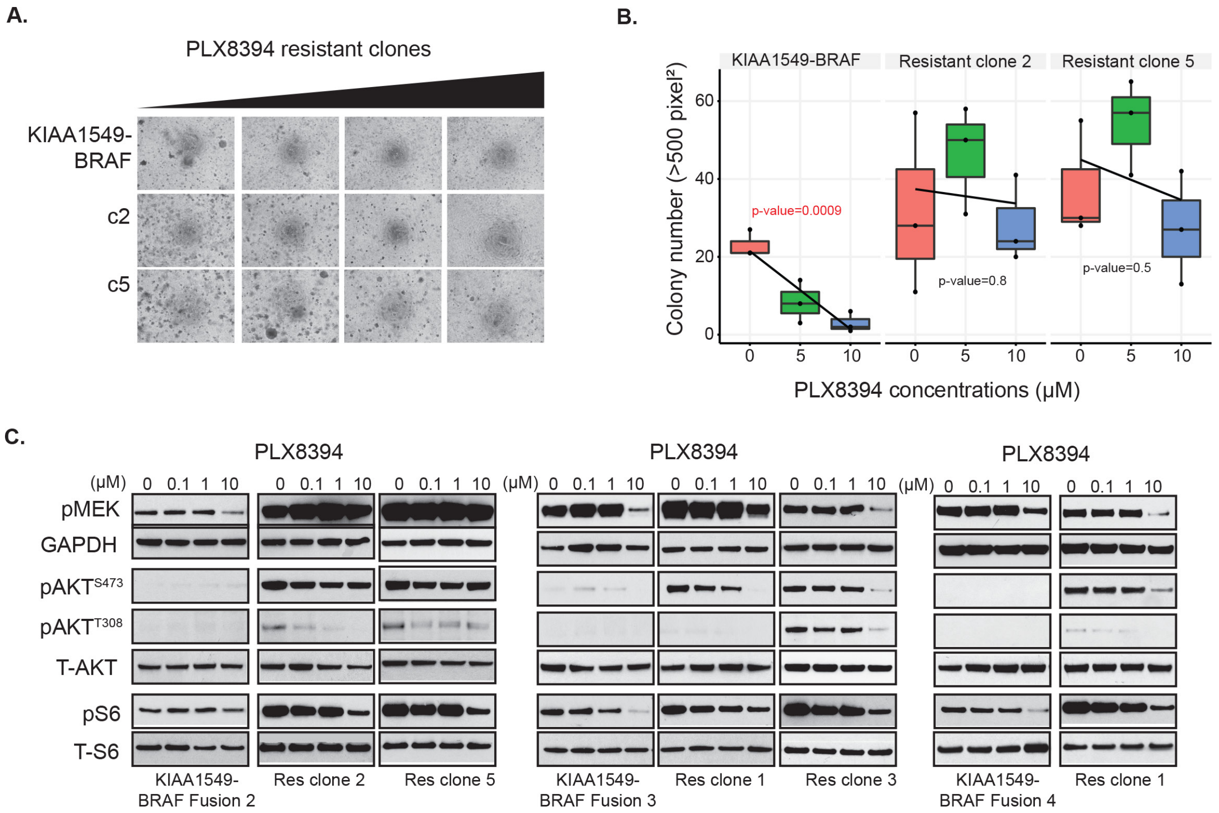

Supplementary Figure 3: PLX8394 resistance demonstrated in three additional KIAA1549-BRAF fusion variants. (A) Representative soft agar images used in analysis of Figure 4A. (B) Linear regression analysis plots for colony numbers shown in figure 4A. (C) Stably expressing KIAA1549-BRAF fusion NIH3T3 parental cells (Fusion-2, Fusion-3, and Fusion-4 as previously characterized) and PLX8394 resistant cells incubated in increasing concentrations of PLX8394 and lysates were immunoblotted and probed as indicated.

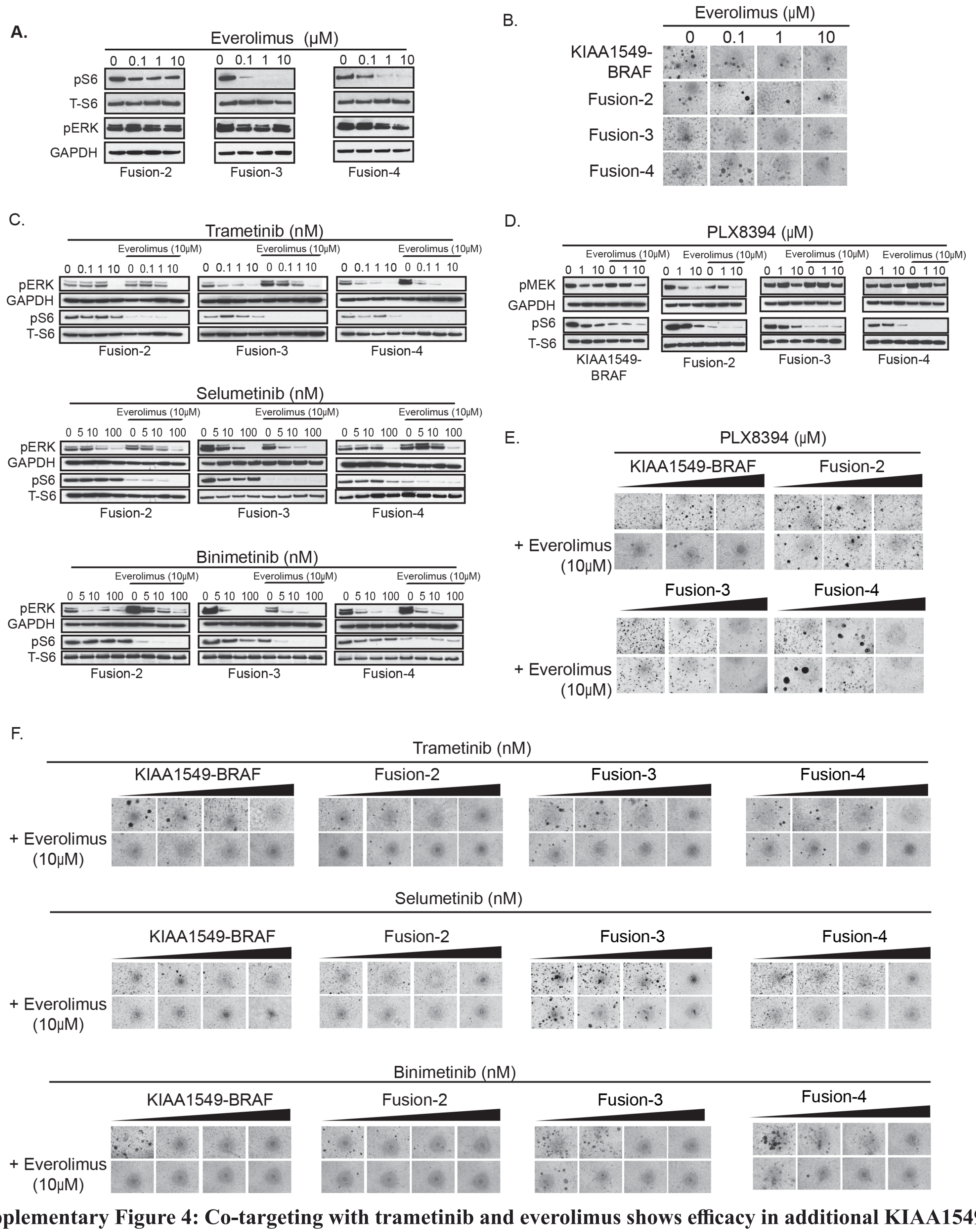

Supplementary Figure 4: Co-targeting with trametinib and everolimus shows efficacy in additional KIAA1549-BRAF variants. (A) KIAA1549-BRAF variants (Fusion-2, Fusion-3, and Fusion-4) expressed in NIH3T were incubated with increasing concentrations of everolimus and cell lysates were immuno-blotted and probed. (B) Soft afar assay using increasing concentrations of everolimus against KIAA1549-BRAF variants (Fusion-2, Fusion-3, and Fusion-4). (C) KIAA1549-BRAF fusion-2, fusion-3, and fusion-4 lines were plated in increasing concentration of trametinib, selumetinib or binimetinib as single agent and in combination with everolimus. (D) KIAA1549-BRAF fusion lines were plated with increasing concentration of PLX8394 as single agent and with everolimus for signaling assay and (E) soft agar assay. (F) KIAA1549-BRAF fusion variants were assayed for colony formation in soft agar in the presence of increasing concentrations of trametinib and everolimus. Displayed are representative soft agar images used in analysis of Figure 5B. (G) KIAA1549-BRAF expressing cell lines were injected as flank xenografts and mice treated daily with lower doses of trametinib and everolimus as indicated (n=10, SEM values shown).

Supplementary Table 1: Tyrosine kinases display altered expression in the context of MEKi emergent resistance. Gene signature sets for MEKi resistant clones expressing KIAA-BRAF were evaluated for enrichment across the 10 major kinase groups. Tyrosine kinases displayed the most significantly enriched group. (AGC Containing PKA, PKG, PKC families; CAMK Calcium/calmodulin-dependent protein kinase;CK1 Casein kinase 1; CMGC Containing CDK, MAPK, GSK3, CLK families; STE Homologs of yeast Sterile 7, Sterile 11, Sterile 20 kinases; TK Tyrosine kinase; TKL Tyrosine kinase–like; RGC Receptor Guanylate Cyclases).

Group	Total # annotated kinases	Group size	Gene set hit	P-value
**TK**	620	95	15	0.003
**Other**	620	101	3	0.017
**CAMK**	620	113	9	0.151
**AGC**	620	69	4	0.157
**TKL**	620	49	5	0.167
**CMGC**	620	76	6	0.178
**STE**	620	53	4	0.210
**Atypical**	620	39	2	0.216
**CK1**	620	17	1	0.360
**RGC**	620	8	1	0.361
